# Not proper ROC curves as new tool for the analysis of differentially expressed genes in microarray experiments

**DOI:** 10.1186/1471-2105-9-410

**Published:** 2008-10-03

**Authors:** Stefano Parodi, Vito Pistoia, Marco Muselli

**Affiliations:** 1 Epidemiology and Biostatistics Section, Scientific Directorate, G. Gaslini Children's Hospital, Genoa, Italy; 2 Laboratory of Oncology, G. Gaslini Children's Hospital, Genoa, Italy; 3 Institute of Electronics, Computer and Telecommunication Engineering, Italian National Research Council, Genoa, Italy

## Abstract

**Background:**

Most microarray experiments are carried out with the purpose of identifying genes whose expression varies in relation with specific conditions or in response to environmental stimuli. In such studies, genes showing similar mean expression values between two or more groups are considered as not differentially expressed, even if hidden subclasses with different expression values may exist. In this paper we propose a new method for identifying differentially expressed genes, based on the area between the ROC curve and the rising diagonal (*ABCR*). *ABCR *represents a more general approach than the standard area under the ROC curve (*AUC*), because it can identify both proper (*i.e.*, concave) and not proper ROC curves (NPRC). In particular, NPRC may correspond to those genes that tend to escape standard selection methods.

**Results:**

We assessed the performance of our method using data from a publicly available database of 4026 genes, including 14 normal B cell samples (NBC) and 20 heterogeneous lymphomas (namely: 9 follicular lymphomas and 11 chronic lymphocytic leukemias). Moreover, NBC also included two sub-classes, *i.e.*, 6 heavily stimulated and 8 slightly or not stimulated samples. We identified 1607 differentially expressed genes with an estimated False Discovery Rate of 15%. Among them, 16 corresponded to NPRC and all escaped standard selection procedures based on *AUC *and *t *statistics. Moreover, a simple inspection to the shape of such plots allowed to identify the two subclasses in either one class in 13 cases (81%).

**Conclusion:**

NPRC represent a new useful tool for the analysis of microarray data.

## Background

Microarray technology allows to analyze the expression of thousands of genes in a single experiment [1]. The identification of genes whose expression changes in pathological conditions or upon exposure to stimuli, such as pharmacologic treatment, is a very common aim of microarray-based studies. In this respect, different statistical tests, generally based on measures of distance between classes, have been so far proposed [2-4]. Among them, two parameters of Receiver Operating Characteristic (ROC) curves, namely the area under the curve (*AUC*), and the partial area at a selected high specificity threshold (*pAUC*), have been applied for such a purpose [4-8]. A ROC curve represents the relationship between the true positive fraction (TPF) and the false positive fraction (FPF) resulting from a set of binary classification tests based on each possible decision threshold value [5,9]. TPF is commonly known as Sensitivity, while FPF corresponds to 1 – Specificity. When a ROC curve is drawn using a specific gene expression profile, *AUC *estimates the probability that a subject randomly selected from one class (*e.g.*, a group of individuals affected by a specific disease) has an expression value higher than a subject randomly selected from the other class (*e.g.*, healthy individuals) [6].

**Figure 1:**
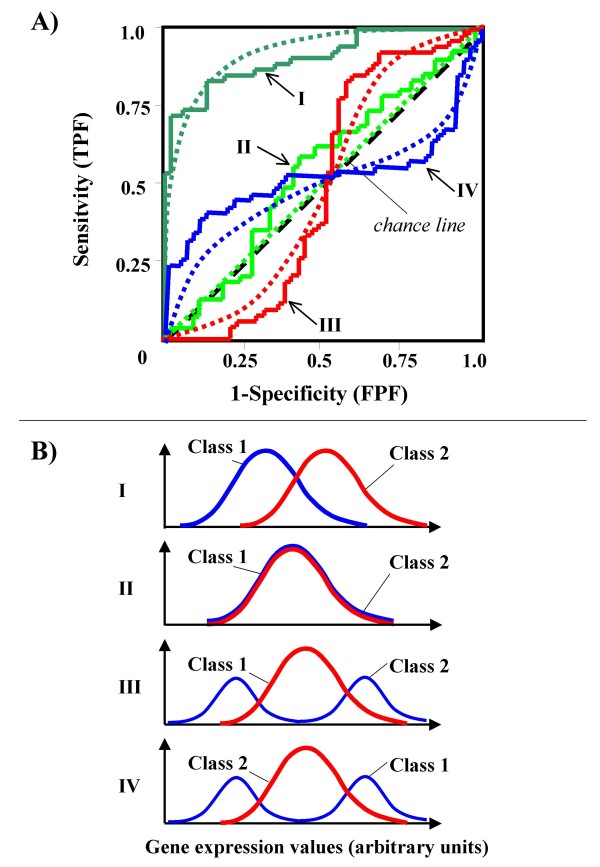
**Theoretical (dotted lines) and empirical (solid lines) ROC curves (panel A) and the corresponding distribution of gene expression values (panel B)**. Empirical ROC curves were obtained using 50 samples randomly selected from each class.

In case of unimodal distributions in the two classes with similar variance and different mean, the corresponding ROC curve tends to lie completely above the diagonal line and to be concave ("proper" ROC curve, *e.g.*, Curve I in Figure 1). In the case of two unimodal distributions with similar mean and variance in the two classes, the corresponding ROC curve will approach the rising diagonal (Curve II in Figure 1). This particular ROC curve is often named the "*chance line*", because it represents the set of all the possible statistical tests with equal probability for a true positive and a false positive result, *i.e.*, corresponding to the set of results expected by chance alone. In such a case *AUC *will tend to 0.5. However, *AUC *values close to 0.5 may also be obtained from genes differentially expressed among two classes, when the presence of a hidden bimodal or multimodal distribution in either class causes the ROC curve to cross the *chance line *[10], like the not concave (not proper) Curve III and Curve IV in Figure 1. Bimodal or multi-modal distributions within a class may indicate the presence of unknown subclasses with different expression values [10]. As a consequence, the identification of such subclasses may provide useful insights about biological mechanisms underlying physiologic or pathologic conditions. However, most expression profiles corresponding to not proper ROC curves (NPRC) are likely to be discharged by the commonly used feature selection methods (including *AUC*, *pAUC *and Student's *t *statistics), because either mean or median values tend to be similar between the considered groups.

To allow the identification of different kind of differentially expressed genes, we have developed a new statistical method of feature selection based on the area between the ROC curve and the rising diagonal (*ABCR*). Furthermore, to separate NPRC (like Curve III and Curve IV in Figure 1) from both uninformative and proper ROC plots (like Curve II and Curve I, respectively) we have developed a new approach based on the combination of standard feature selection procedures based either on *AUC* or on *t* test with a new statistical test based on a simple variant of *ABCR* (*TNRC* = Test for Not-proper ROC Curves).

The performance of our method was evaluated by comparing the gene expression profiles in two different classes, using data from a publicly available data base including 4026 gene expression profiles [11]. Class A included 14 different samples of normal circulating B cells (NBC), class B included 20 heterogeneous lymphomas. Class A and B both included two subclasses, namely: 6 heavily stimulated and 8 slightly stimulated or unstimulated samples in class A (Table 1); 9 follicular lymphomas (FL) and 11 chronic lymphocytic leukemias (CLL) in class B.

**Table 1:** Pattern of stimulation of the 14 normal circulating B cells in class A

N	Pattern of stimulation
	*Heavily stimulated cells*
1	Blood B cells;anti-IgM+CD40L low 48 h
2	Blood B cells;anti-IgM+CD40L high 48 h
3	Blood B cells;anti-IgM+CD40L 24 h
4	Blood B cells;anti-IgM 24 h
5	Blood B cells;anti-IgM+IL-4 24 h
6	Blood B cells;anti-IgM+CD40L+IL-4 24 h
	*Slightly or not stimulated cells*
7	Blood B cells;anti-IgM+IL-4 6 h
8	Blood B cells;anti-IgM 6 h
9	Blood B cells;anti-IgM+CD40L 6 h
10	Blood B cells;anti-IgM+CD40L+IL-4 6 h
11	Blood B cells;memory CD27+
12	Blood B cells;naive CD27-
13	Blood B cells
14	Cord Blood B

Note: sample number (N) corresponds to the original location of each sample in the original data set: .

The aim of this study is to illustrate a new comprehensive approach based on the combination of both standard (*AUC*) and new (*ABCR *and *TNRC*) ROC parameters. Moreover, we show how not proper ROC curves, identified by *TNRC*, may allow at the same time both to select differentially expressed genes that tend to escape standard statistical tools, and to point out the presence of hidden subclasses with biological or clinical meaning. For such purposes, we selected the genes with the highest *ABCR *value corresponding to an *a priori *chosen False Discovery Rate (FDR) [12]. Among the expression profiles selected by *ABCR *we identified over-expressed and under-expressed genes using either the Area Under the ROC curve (*AUC*) or the Student's *t *statistic, which both represent standard methods for feature selection in microarray analysis [3,4,8]. NPRC were identified by high values of *TNRC *statistic. A conventional unadjusted p value of 0.05 was used as threshold in each analysis. Furthermore, we conducted a detailed analysis of each selected NPRC, to assess the concordance between the observed gene expression and the presence of hidden subclasses (see Material and Methods for more details).

The FDR of both standard (*AUC *and *t *value) and new proposed statistics (*ABCR *and *TNRC*) was also estimated under some different distribution hypotheses and at different sample size by using artificial data sets containing 4000 simulated gene expression profiles in two classes. Finally, the distribution of *ABCR *and *TNRC *at some different sample size under the null hypothesis of no differentially expressed genes between two classes was estimated by extensive random permutation analysis.

## Results

We grouped all the genes discussed below as follows: lymphocyte/macrophage related genes (group 1), major histocompatibility complex related genes (group 2), genes involved in malignant cell transformation (group 3), genes related to nucleic acid metabolism or DNA transcription (group 4), and gene encoding various enzymes/kinases and other proteins (group 5). In spite of some overlap, this classification allows to subdivide the tested genes according to their functional features.

**Figure 2:**
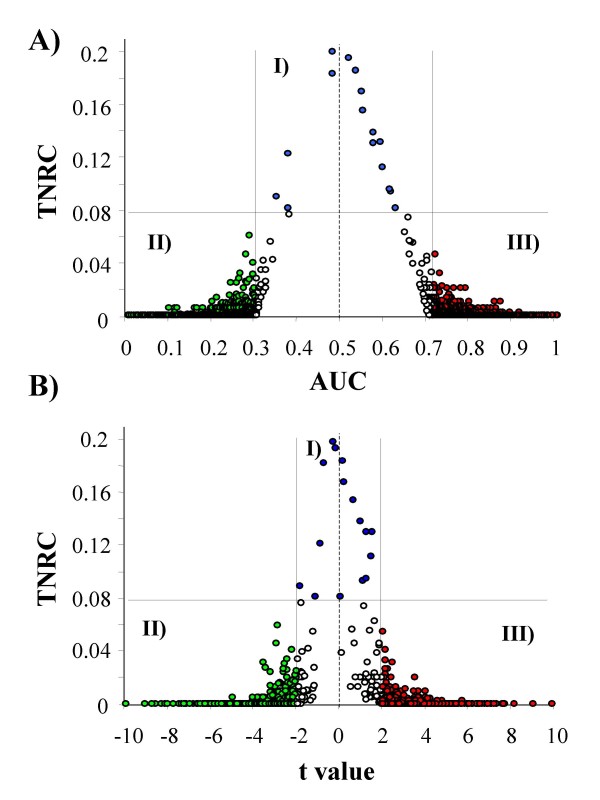
***TNRC *value for the 1607 top genes selected by *ABCR *at FDR = 15%, as a function of *AUC *(Panel A) and *t *statistics (Panel B)**. Area I includes genes corresponding to not proper ROC curves (blue circles); Area II includes genes under-expressed in malignant cells (green circles); Area III includes genes over-expressed in malignant cells (red circles); empty circles correspond to unselected genes. Solid lines represent the thresholds corresponding to p = 0.05 for *TNRC *(horizontal line in Panel A and in Panel B), for *AUC *(vertical lines in Panel A) and for *t *statistic (vertical lines in Panel B). Broken lines represent the expected value under the null hypothesis for *AUC *(Panel A) and for *t *statistics (Panel B).

Our method identified 1607 genes with the highest value of *ABCR *at a selected FDR of 15%. The estimated selection probability ranged from 40% to 100% (median value: 79.5%). Figure 2A shows the results of the selection procedure combining the new proposed *TNRC *statistic with the standard *AUC *on the subset of 1607 genes selected by *ABCR*. Area I includes 16 genes with a statistically significant *TNRC *value (blue circles), while areas II and III collect 1524 genes with a statistically significant *AUC *value. In particular, genes in area II (green circles) were found to be under-expressed in class A compared with class B, while genes in area III (red circles) were found to be over-expressed in class A. No genes corresponding to NPRC were identified by *AUC *statistic and *vice versa*. However, 67 genes (empty circles in Figure 2A) were not identified by either *AUC *or *TNRC*. The large majority of them (n = 57) had a borderline statistically significant value of *AUC *(p value between 0.05 and 0.10), but not for *TNRC*, while 2 genes had a borderline p value for *TNRC*, but not for *AUC*. The analysis was repeated using *t *statistic in place of *AUC*, and the corresponding results are summarized in Figure 2B. Also in this case, no gene with statistically significant *TNRC *value was identified by the standard statistical method (*i.e.*, *t *value). However, the separation between this two different kind of differentially expressed genes was less evident than that obtained by using *AUC *(Figure 2A). Moreover, a larger number of genes (n = 112) remained unclassified (empty circles), including some genes with a very low value for both *TNRC *and *t *statistics.

Table 2 shows the 16 genes with the highest *TNRC *value, corresponding to the blue circles in Figure 2A and 2B. Among them, 4 had an unknown function. The others belonged to group 1 (genes n. 1–3, and n.13), group 3 (genes n. 4 and, 8), group 4 (gene n. 5), or group 5 (genes n. 6, 7, 11, 12, and 15) [13]. Values of *TNRC *parameter ranged from 0.082 to 0.2, while the corresponding *ABCR *values ranged between 0.202 and 0.253. Interestingly, the first three selected genes in Table 2 included all the clones of the gene for Immunoglobulin J chain in the original data set. Furthermore, the gene n. 6 (*VRK2 kinase*) was present in another clone in the same data set (gene n. 12).

**Table 2:** Comparison between 14 NBC and 20 heterogeneous lymphomas – genes selected by *ABCR *and *TNRC *statistics

N	Gene ID	Gene name	*ABCR*	*TNRC*	Group
1	GENE3389X	*Immunoglobulin J chain*	0.2250	0.2000	1
2	GENE3390X	*Immunoglobulin J chain*	0.2096	0.1954	1
3	GENE3388X	*Immunoglobulin J chain*	0.2143	0.1858	1
4	GENE3323X	*BCL7A*	0.2069	0.1836	3
5	GENE3407X	*Histone deacetylase 3*	0.2122	0.1694	4
6	GENE75X	*VRK2 kinase*	0.2015	0.1552	5
7	GENE1141X	*MAPKKK5*	0.2105	0.1390	5
8	GENE1817X	*BL34*	0.2171	0.1314	3
9	GENE2395X	*Unknown*	0.2025	0.1310	Unknown
10	GENE2696X	*Unknown*	0.2531	0.1224	Unknown
11	GENE3521X	*Similar to KIAA0050*	0.2051	0.1122	5
12	GENE74X	*VRK2 kinase*	0.2043	0.0954	5
13	GENE2287X	*MRC OX-2*	0.2046	0.0940	1
14	GENE3541X	*Unknown*	0.2436	0.0900	Unknown
15	GENE1362X	*Syndecan-2*	0.2031	0.0816	5
16	GENE2673X	*Unknown*	0.2034	0.0816	Unknown

Group 1: lymphocyte/macrophage related genes; Group 2: major histocompatibility complex related genes; Group 3: genes involved in malignant cell transformation; Group 4: genes related to nucleic acid metabolism or DNA transcription; Group 5: genes encoding various enzymes/kinases and other proteins. *ABCR*: Area between the ROC curve and the rising diagonal; *TNRC*: Test for not-proper ROC curves.

Table 3 shows the 16 top genes selected by the standard ROC analysis based on *AUC *values among the 1607 genes reported in Figure 2A. Genes were sorted on the basis of the corresponding pure accuracy (*i.e.*, the probability to correctly rank two samples, one randomly extracted from class A and one from class B), which is estimated by *AUC *for proper curves lying above the *chance line*, and by 1 – *AUC *for proper curves lying below. Genes in Table 3 also corresponded to the 16 highest values of *ABCR*. Eleven genes showed an *AUC *< 0.5 and they were accordingly considered as under-expressed in FL, CLL in comparison with NBC, while the remaining 5 genes showed an *AUC *> 0.5 and were considered as over-expressed. Among the under-expressed genes two belonged to group 1 (genes n. 9 and n. 15), 1 to group 3 (gene n.5), 4 to group 4 (genes n. 2, n. 4, n. 6 and n.14), 3 to group 5 (Genes n.3, n. 8 and n. 16), and 1 had an unknown function (gene n. 13). Among the over-expressed genes, 1 belonged to group 1 (gene n. 7), 1 to group 4 (gene n. 12), and 3 had unknown functions (genes n. 1, n. 10, and n. 11) [13]. *AUC *ranged between 0 and 0.02 in under-expressed genes, and between 0.986 and 1 in over-expressed genes. *ABCR *values ranged between 0.479 and 0.5.

**Table 3:** Comparison between 14 NBC and 20 heterogeneous lymphomas – top 16 genes selected by *ABCR *and *AUC *statistics

N	Gene ID		Gene name	*ABCR*	*AUC*	Group
1	GENE2495X	+	*Unknown*	0.5000	1.0000	*Unknown*
2	GENE1217X	-	*NFkB2*	0.5000	0.0000	4
3	GENE1602X	-	*protein kinase (zpk)*	0.5000	0.0000	5
4	GENE1191X	-	*CREM*	0.5000	0.0000	4
5	GENE1171X	-	*Similar to spi-1*	0.4964	0.0036	3
6	GENE1219X	-	*IkB alpha*	0.4964	0.0036	4
7	GENE3795X	+	*AIM2*	0.4964	0.9964	1
8	GENE1730X	-	*Sgk*	0.4929	0.0071	5
9	GENE1170X	-	*CD83*	0.4929	0.0071	1
10	GENE3702X	+	*Unknown*	0.4893	0.9893	*Unknown*
11	GENE2494X	+	*Unknown*	0.4857	0.9857	*Unknown*
12	GENE3280X	+	*eIF-2B alpha subunit*	0.4857	0.9857	4
13	GENE1160X	-	*Unknown*	0.4857	0.0143	*Unknown*
14	GENE589X	-	*eIF-3*	0.4821	0.0179	4
15	GENE1172X	-	*CD83*	0.4786	0.0214	1
16	GENE324X	-	*Nak1*	0.4786	0.0214	5

Group 1: lymphocyte/macrophage related genes; Group 2: major histocompatibility complex related genes; Group 3: genes involved in malignant cell transformation; Group 4: genes related to nucleic acid metabolism or DNA transcription; Group 5: genes encoding various enzymes/kinases and other proteins. *ABCR*: Area between the ROC curve and the rising diagonal; *AUC *= Area under the ROC curve; "+" = overexpressed; "-" = underexpressed.

All the analyses were repeated varying the FDR threshold. At FDR = 10%, 1454 genes were identified by *ABCR*. Among them, 1439 (99%) were called over- or under-expressed on the basis of *AUC *statistic, 4 genes corresponding to NPRC were identified by *TNRC *and 11 were not identified by either statistic. Among these latter, 8 had a borderline statistical significant value for *AUC *and 1 for *TNRC*. Also in this case, no genes identified by *AUC *were also selected by *TNRC *and *vice versa*. Finally, using FDR = 20% for *ABCR*, 1866 genes were selected. Among them, 1524 (82%) were selected by *AUC*, 24 by *TNRC*, and 318 remained not classified, including 272 genes with a borderline statistical significance for *AUC *and 3 for *TNRC*. Also in this case no genes were identified as differentially expressed by both *AUC *and *TNRC*.

**Figure 3:**
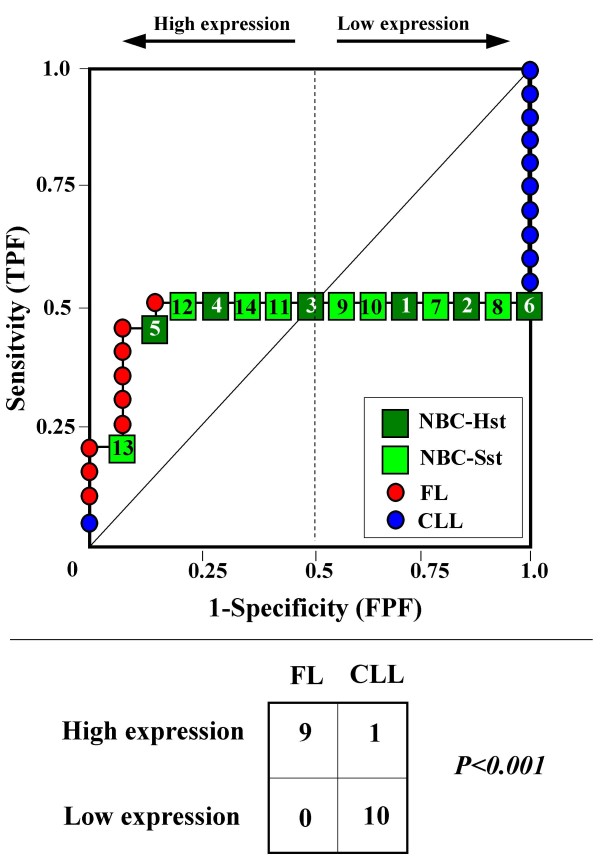
**Not proper ROC curve corresponding to the expression of gene n. 1 in Table 2 (GENE3389X: *Immunoglobulin J Chain*).** Comparison between class A (14 samples of NBC) and class B (20 heterogeneous lymphomas, including 9 FL and 11 CLL samples). Hst = Highly stimulated NBC; SSt= Slightly or not stimulated NBC (Table 1). NBC samples are numbered according to Table 1.

**Figure 4:**
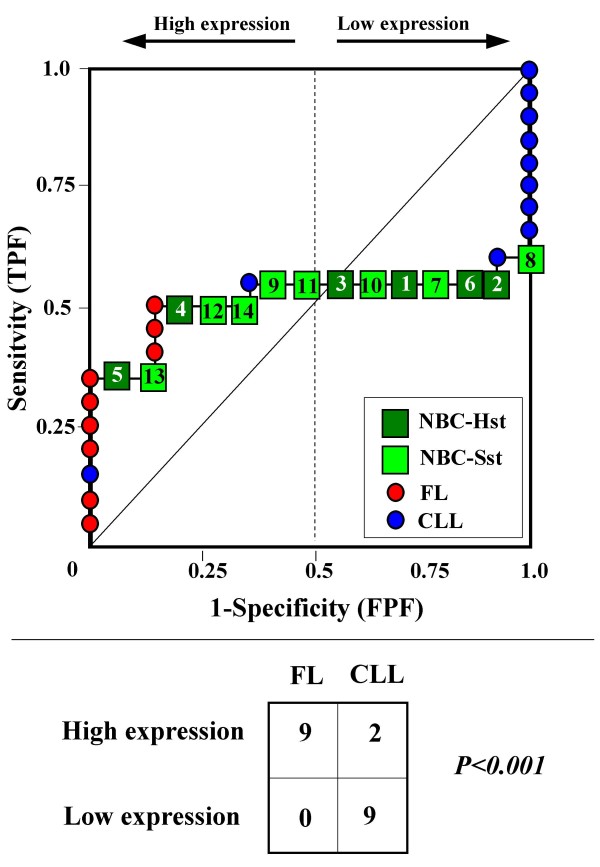
**Not proper ROC curve corresponding to the expression of gene n. 2 in Table 2 (GENE3390X: *Immunoglobulin J Chain*).** Comparison between class A (14 samples of NBC) and class B (20 heterogeneous lymphomas, including 9 FL and 11 CLL samples). Hst = Highly stimulated NBC; SSt= Slightly or not stimulated NBC (Table 1). NBC samples are numbered according to Table 1.

**Figure 5:**
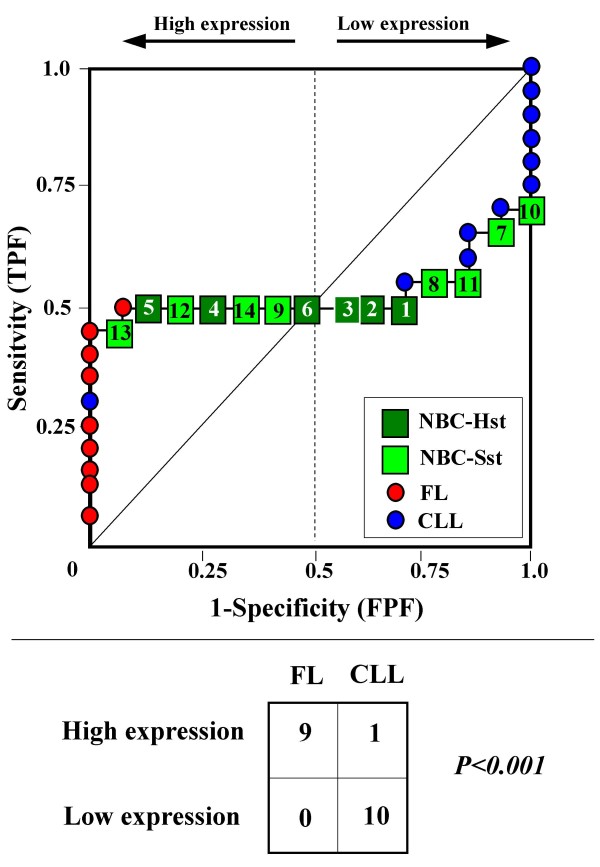
**Not proper ROC curve corresponding to the expression of gene n. 3 in Table 2 (GENE3388X: *Immunoglobulin J Chain*).** Comparison between class A (14 samples of NBC) and class B (20 heterogeneous lymphomas, including 9 FL and 11 CLL samples). Hst = Highly stimulated NBC; SSt= Slightly or not stimulated NBC (Table 1). NBC samples are numbered according to Table 1.

**Figure 6:**
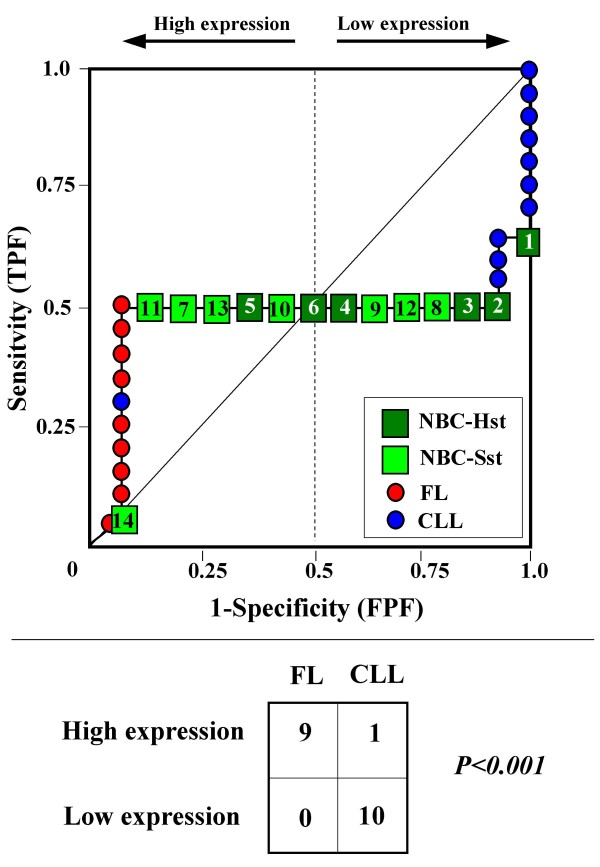
**Not proper ROC curve corresponding to the expression of gene n. 4 in Table 2 (GENE3323X: *BCL7A*).** Comparison between class A (14 samples of NBC) and class B (20 heterogeneous lymphomas, including 9 FL and 11 CLL samples). Hst = Highly stimulated NBC; SSt= Slightly or not stimulated NBC (Table 1). NBC samples are numbered according to Table 1.

**Figure 7:**
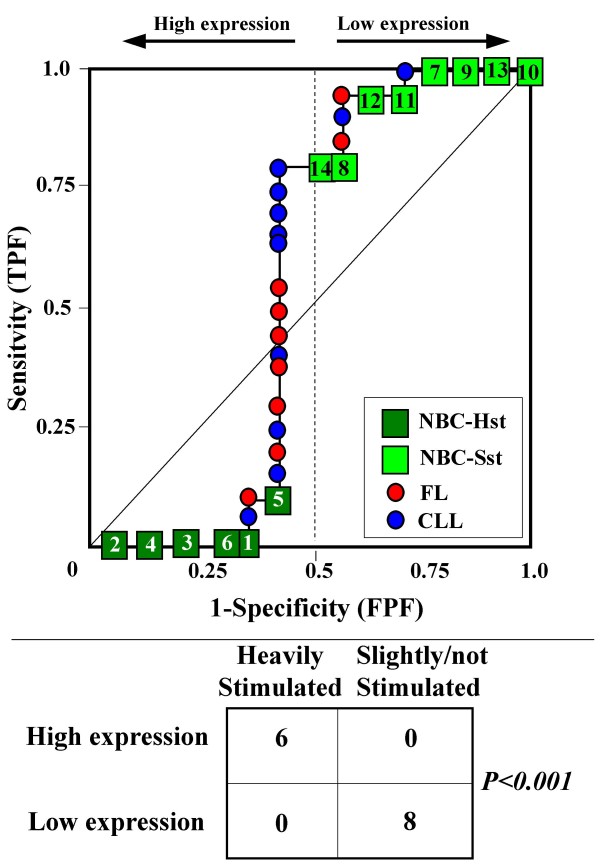
**Not proper ROC curve corresponding to the expression of gene n. 5 in Table 2 (GENE3407X: *Histone deacetylase 3*).** Comparison between class A (14 samples of NBC) and class B (20 heterogeneous lymphomas, including 9 FL and 11 CLL samples). Hst = Highly stimulated NBC; SSt = Slightly or not stimulated NBC (Table 1). NBC samples are numbered according to Table 1.

**Figure 8:**
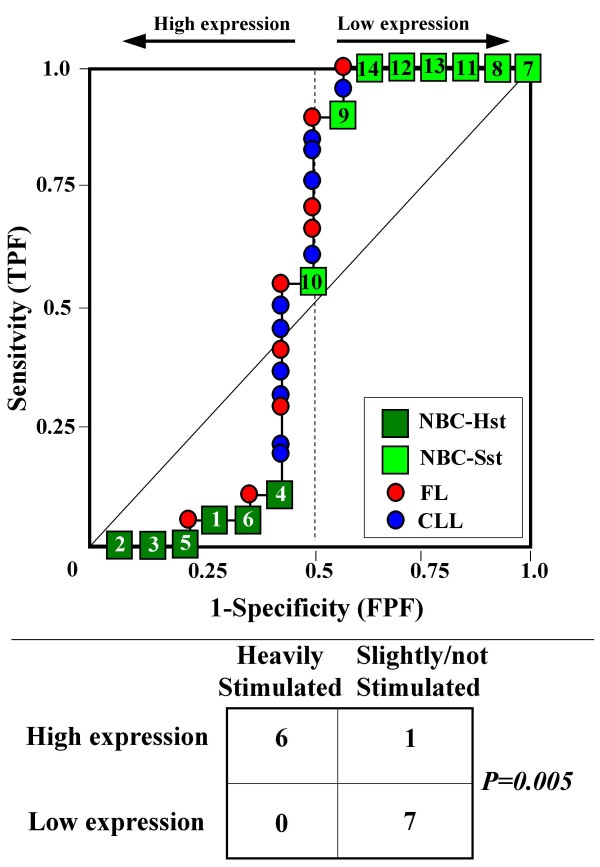
**Not proper ROC curve corresponding to the expression of gene n. 6 in Table 2 (GENE75X: *VRK2 kinase*).** Comparison between class A (14 samples of NBC) and class B (20 heterogeneous lymphomas, including 9 FL and 11 CLL samples). Hst = Highly stimulated NBC; SSt= Slightly or not stimulated NBC (Table 1). NBC samples are numbered according to Table 1.

**Figure 9:**
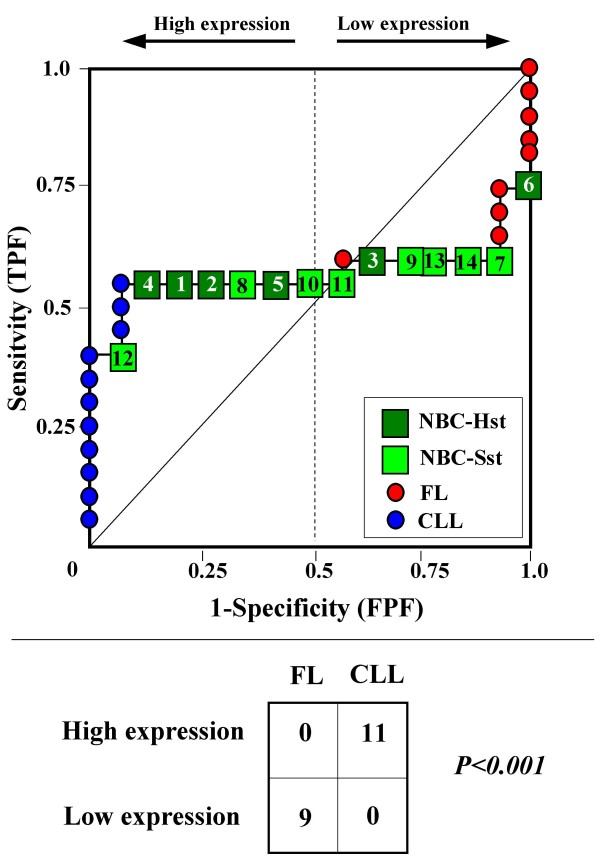
**Not proper ROC curve corresponding to the expression of gene n. 7 in Table 2 (GENE1141X: *MAPKKK5*).** Comparison between class A (14 samples of NBC) and class B (20 heterogeneous lymphomas, including 9 FL and 11 CLL samples). Hst = Highly stimulated NBC; SSt= Slightly or not stimulated NBC (Table 1). NBC samples are numbered according to Table 1.

**Figure 10:**
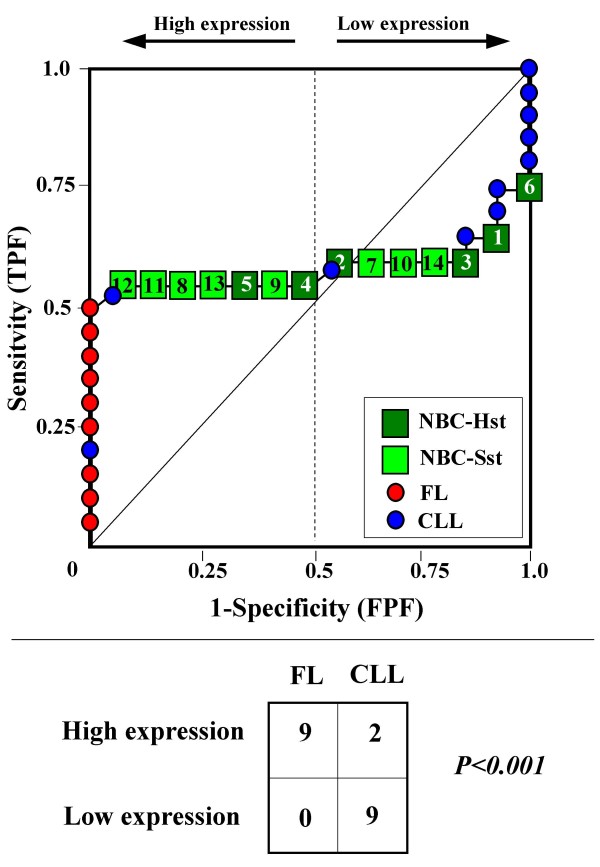
**Not proper ROC curve corresponding to the expression of gene n. 8 in Table 2 (GENE1817X: *BL34*).** Comparison between class A (14 samples of NBC) and class B (20 heterogeneous lymphomas, including 9 FL and 11 CLL samples). Hst = Highly stimulated NBC; SSt= Slightly or not stimulated NBC (Table 1). NBC samples are numbered according to Table 1.

**Figure 11:**
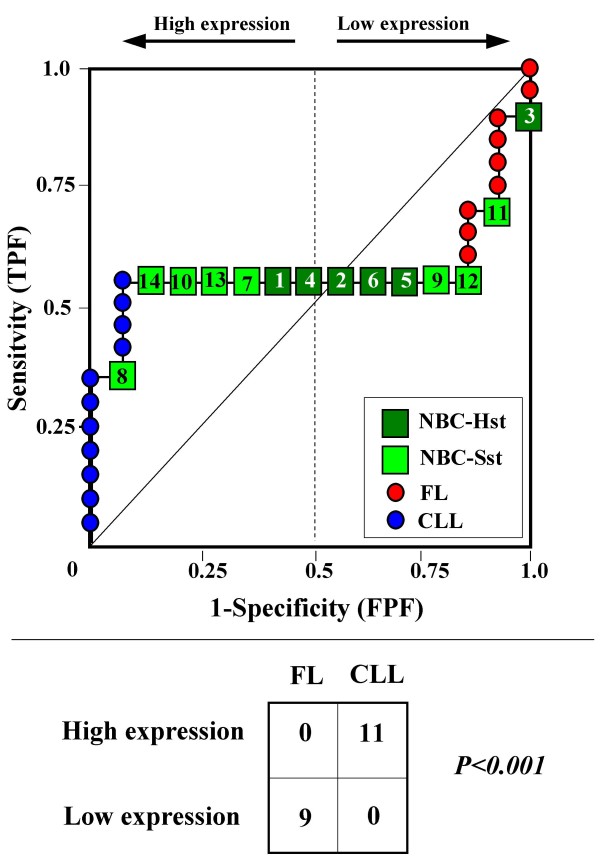
**Not proper ROC curve corresponding to the expression of gene n. 9 in Table 2 (GENE2395X: *unknown*).** Comparison between class A (14 samples of NBC) and class B (20 heterogeneous lymphomas, including 9 FL and 11 CLL samples). Hst = Highly stimulated NBC; SSt= Slightly or not stimulated NBC (Table 1). NBC samples are numbered according to Table 1.

**Figure 12:**
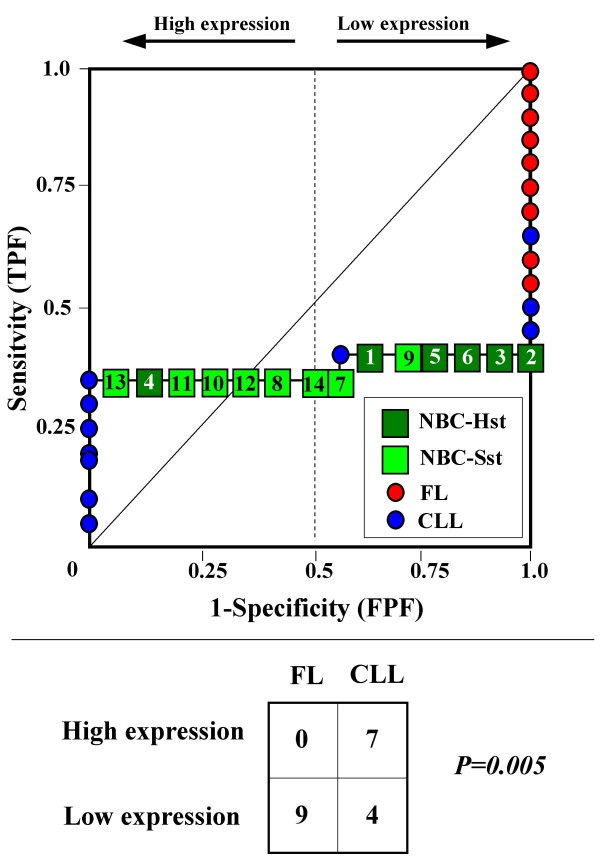
**Not proper ROC curve corresponding to the expression of gene n. 10 in Table 2 (GENE2696X: *unknown*).** Comparison between class A (14 samples of NBC) and class B (20 heterogeneous lymphomas, including 9 FL and 11 CLL samples). Hst = Highly stimulated NBC; SSt= Slightly or not stimulated NBC (Table 1). NBC samples are numbered according to Table 1.

**Figure 13:**
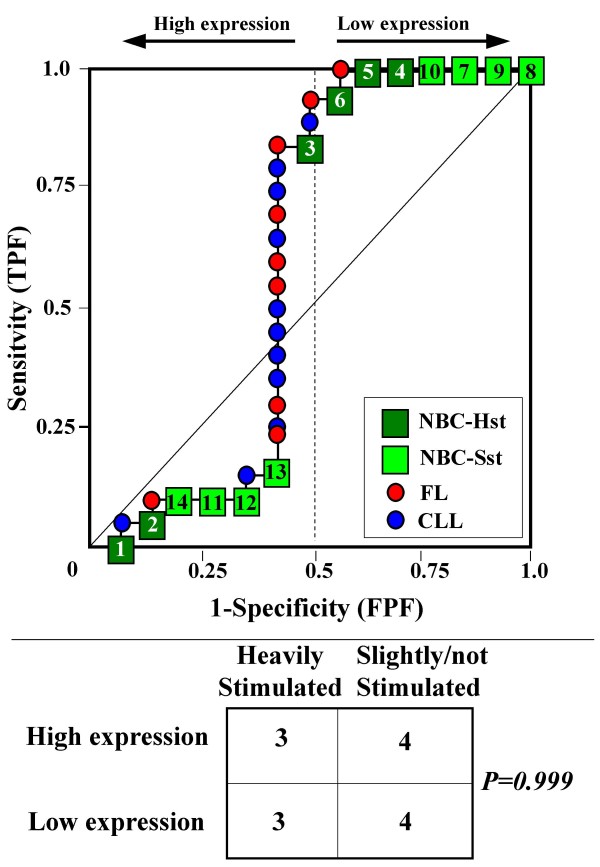
**Not proper ROC curve corresponding to the expression of gene n. 11 in Table 2 (GENE3521X: *Similar to KIAA0050*).** Comparison between class A (14 samples of NBC) and class B (20 heterogeneous lymphomas, including 9 FL and 11 CLL samples). Hst = Highly stimulated NBC; SSt= Slightly or not stimulated NBC (Table 1). NBC samples are numbered according to Table 1.

**Figure 14:**
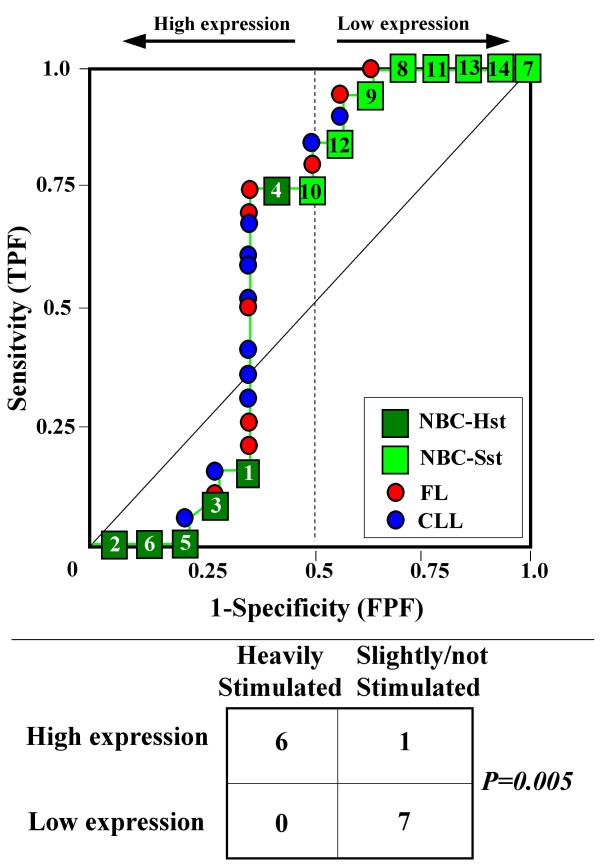
**Not proper ROC curve corresponding to the expression of gene n. 12 in Table 2 (GENE74X: *VRK2 kinase*).** Comparison between class A (14 samples of NBC) and class B (20 heterogeneous lymphomas, including 9 FL and 11 CLL samples). Hst = Highly stimulated NBC; SSt= Slightly or not stimulated NBC (Table 1). NBC samples are numbered according to Table 1.

**Figure 15:**
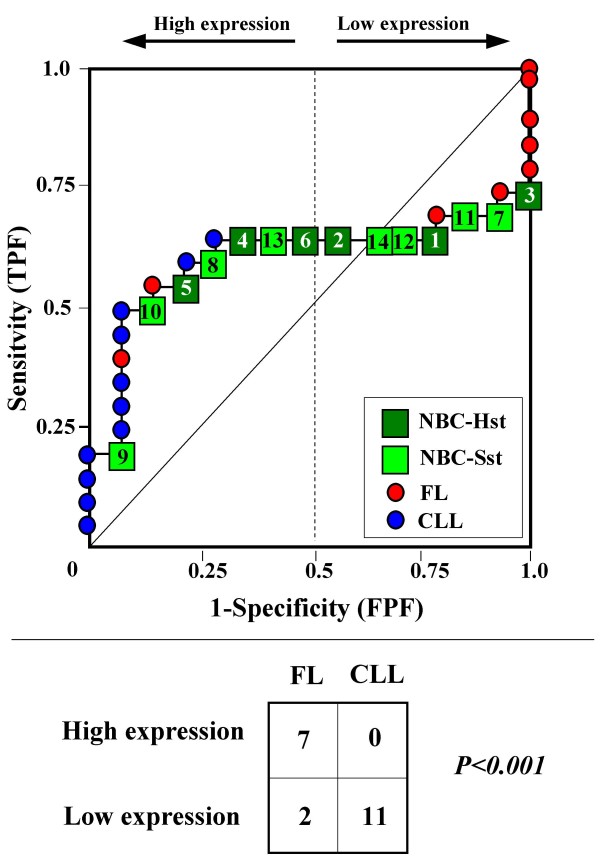
**Not proper ROC curve corresponding to the expression of gene n. 13 in Table 2 (GENE2287X: *MRC OX-2*).** Comparison between class A (14 samples of NBC) and class B (20 heterogeneous lymphomas, including 9 FL and 11 CLL samples). Hst = Highly stimulated NBC; SSt= Slightly or not stimulated NBC (Table 1). NBC samples are numbered according to Table 1.

**Figure 16:**
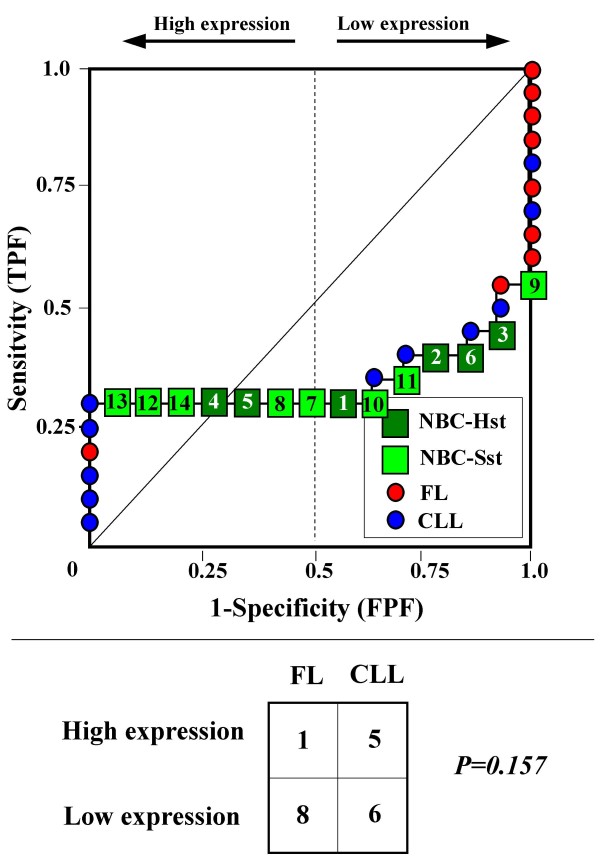
**Not proper ROC curve corresponding to the expression of gene n. 14 in Table 2 (GENE3541X: *Unknown*).** Comparison between class A (14 samples of NBC) and class B (20 heterogeneous lymphomas, including 9 FL and 11 CLL samples). Hst = Highly stimulated NBC; SSt= Slightly or not stimulated NBC (Table 1). NBC samples are numbered according to Table 1.

**Figure 17:**
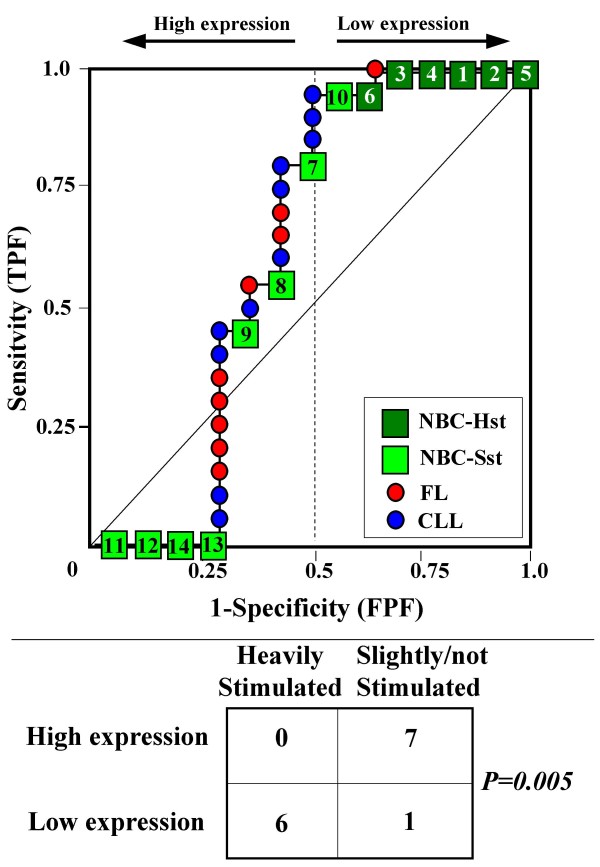
**Not proper ROC curve corresponding to the expression of gene n. 15 in Table 2 (GENE1362X: *Syndecan-2*).** Comparison between class A (14 samples of NBC) and class B (20 heterogeneous lymphomas, including 9 FL and 11 CLL samples). Hst = Highly stimulated NBC; SSt= Slightly or not stimulated NBC (Table 1). NBC samples are numbered according to Table 1.

**Figure 18:**
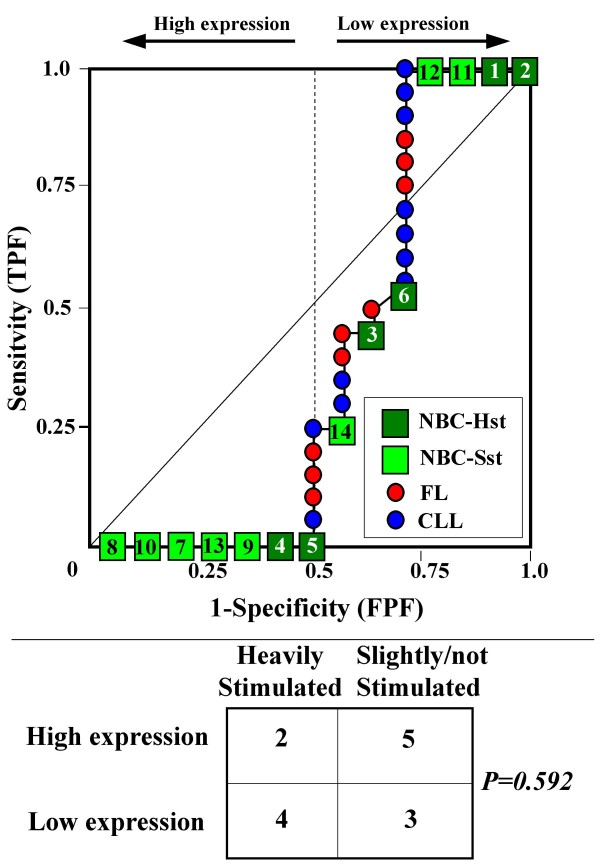
**Not proper ROC curve corresponding to the expression of gene n. 16 in Table 2 (GENE2673X: *Unknown*).** Comparison between class A (14 samples of NBC) and class B (20 heterogeneous lymphomas, including 9 FL and 11 CLL samples). Hst = Highly stimulated NBC; SSt= Slightly or not stimulated NBC (Table 1). NBC samples are numbered according to Table 1.

Because the main sources of heterogeneity were known *a priori *for both class A (NBC), which included differently stimulated cells, and for class B, which included samples from two different malignant diseases (namely, FL and CLL), we carried out a detailed analysis of each ROC curve obtained from the expression values of genes listed in Table 2 (Figures 3, 4, 5, 6, 7, 8, 9, 10, 11, 12, 13, 14, 15, 16, 17 and 18). Figures from 3 to 18 were ordered according to the ranks of the corresponding genes in Table 2, *i.e.*, Figure 3 refers to the expression of gene n. 1, Figure 4 corresponds to gene n. 2, and so on. Each plot reports both the origin of samples in class B (*i.e.*, either FL or CLL) and the two major subclasses within NBC class, according to Table 1 (*i.e.*, heavily stimulated and slightly or not stimulated cells). Moreover, each plot was arbitrarily split into two parts to roughly separate samples with high (left side) and with low (right side) expression level. Finally, the ROC curves in Figures 3, 4, 5, 6, 7, 8, 9, 10, 11, 12, 13, 14, 15, 16, 17. 18 were classified as "sigmoid-shaped" (like Curve III in Figure 1) and "inversely sigmoid-shaped" (like Curve IV in Figure 1).

Six out of the 16 genes in Table 2 corresponded to sigmoid-shaped curves (Figures 7, 8, 13, 14, 17, and 18). Four curves allowed to identify the two hidden subgroups of highly stimulated and slightly or not stimulated B cells, with no error for Figure 7 (corresponding to the expression of *Histone deacetylase*, gene n. 5 in Table 2), 1 error for Figures 8, 14 (both corresponding to clones of *VRK2 kinase*, genes n. 6 and n. 12, respectively) and 17 (corresponding to *Syndecan-2*, gene n. 15). All the remaining 10 genes in Figure 3 corresponded to inversely-sigmoid shaped curves and they allowed to separate the two hidden subclasses within class B (FL and CLL), with the only exception of Figure 16 (gene n.14, unknown function). In more detail, the identification of the two hidden subclasses was made with no error in 2 cases (Figure 9, corresponding to gene *MAPKKK5*, n. 7, and Figure 11, gene n. 9 with unknown function), with 1 error in 3 cases (Figure 3 and Figure 5, corresponding to two clones of *Immunoglobulin J chain*, genes n.1 and n.3, respectively, and Figure 6, *BCL7A*, gene n. 4), with 2 errors in 3 cases (Figure 4, gene n. 2, *Immunoglobulin J chain*; Figure 10, gene n. 8, *BL34*; Figure 15, gene n. 13, *MRC OX-2*), and 4 errors in 1 case (Figure 12, gene n. 10, unknown function). In summary, only 3 out of 16 ROC curves (Figures 13, 16 and 18) were not associated with the presence of *a priori *known hidden subclasses.

**Figure 19:**
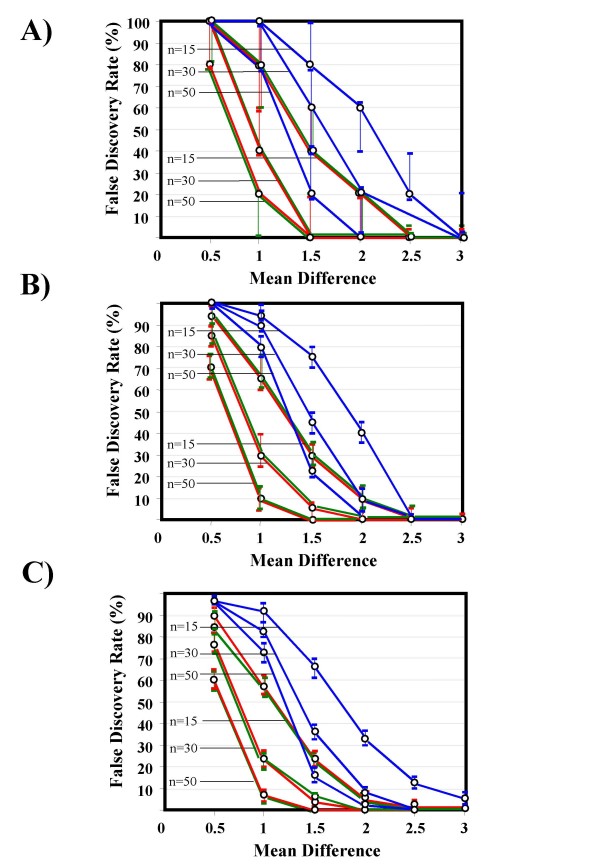
**False Discovery Rate of *ABCR *(green line), *TNRC *(blue line) and *AUC *(red line) as a function of the mean difference between class, the sample size in each class and the number N of selected genes.** Median and interquartile range are displayed. Panel A: N = 5; panel B: N = 20; Panel C: N = 50.

**Figure 20:**
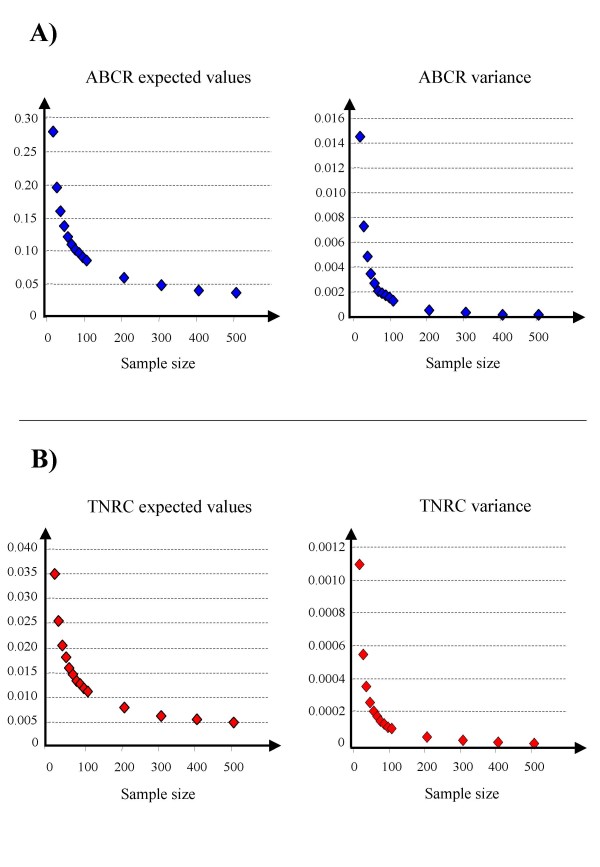
**Mean and variance estimates for *ABCR *and *TNRC *under the null hypothesis as a function of the number of samples in each class (equal sample size).** Each estimate was obtained from 10^4 ^random permutations.

Figure 19 (A-C) shows the results of the analysis of simulated data sets, reporting, for comparison purposes, the performance of *ABCR*, *TNRC* and the commonly used *AUC* statistic. As expected, for all the considered statistics FDR tended to decrease by increasing the sample size, the mean difference (MD) between groups and the number of selected genes. The performance of *ABCR* and *AUC* was similar in each analysis, while FDR estimates were systematically higher for *TNRC* than for the other two statistics. Finally, Figure 20 shows the expected values and variance for the two new proposed ROC parameters (*ABCR*, panel A, and TNRC, panel B) estimated under the null hypothesis by 10^4^ simulations based on a random permutation analysis. For both statistics the estimated expected value tended to the theoretical one (i.e., 0 in both cases) increasing the sample size, while the variance tended to rapidly decrease, indicating that both *ABCR* and *TNRC* are asymptotically unbiased and consistent estimators.

## Discussion

In this paper we have illustrated a new feature selection method using a combination of standard (*AUC*) and new (*ABCR *and *TNRC*) statistical tools based on ROC curves properties. In particular, *ABCR *represents a new comprehensive test to identify both proper and not proper ROC curves. Because *ABCR *is a measure of the distance between the cumulative distributions of the two classes under study (as demonstrated in the Material and Methods section), it may be used to potentially identify any kind of differentially expressed genes. *AUC *represents a well known useful tool to identify under- and over-expressed genes in the comparison between two classes [4,7,8,12], while *TNRC *represents a new tool to specifically identify NPRC. As illustrated in Figure 2A, where genes selected by *ABCR *were separated on the basis of *AUC *and *TNRC *values, all the genes identified by *TNRC *tended to escape feature selection based on *AUC *and *vice versa*. This behavior was also confirmed when *AUC *was replaced by *t *statistic, another standard feature selection tool [3,4], and when two different thresholds for FDR were used (10% and 20%, respectively). These results strongly point out that *TNRC *can identify differentially expressed genes that are hardly identifiable by standard statistical tools.

The large majority of genes selected by *ABCR *were identifiable by *AUC *or *t *statistic and, accordingly, they resulted either under- or over-expressed in lymphoma cells compared with NBC. NPRC represented a very small fraction of the selected genes. This finding might be due to the fact that *TNRC *statistic tends to identify gene expression profiles that are different in two or more subclasses within one class compared with another, a condition that may be quite rare in real data. However, as indicated by results of simulation analysis (Figure 19) the main limit of *TNRC *is probably its low statistical power. Simulation analysis was based on a very simplified scenario, because data were generated from a few variety of statistical distributions (namely: normal and bi-normal functions, with similar variance and different means) and the correlation between gene expression profiles was not taken into account. However, in spite of such limitations, the comparison between *TNRC, ABCR *and *AUC *clearly indicated a poor performance of *TNRC *compared with the two latter statistics (Figure 19). As a consequence, in microarray experiments with small sample size *TNRC *can probably recover only a minor proportion of differentially expressed genes that have escaped standard feature selection methods. However, as illustrated in Figure 2A and 2B, *TNRC *represents a complementary tool for microarray data that may supplement information from standard statistical analysis. Moreover, the rapid improvements in microarray technology and the consequent availability of chips with a low cost and a high quality might allow a very extensive application of *TNRC *method in the next future.

We have arbitrarily chosen the conventional threshold of p = 0.05 to separate different kinds of gene expression profiles (Figure 2A and 2B). It is evident from figure 2A, where only ROC parameters were used, that varying the selected thresholds, almost all unselected genes may have been included in one out of the three considered categories (namely: under-expressed, over-expressed and corresponding to NPRC, respectively). Using *t *statistic in place of *AUC *(Figure 2B) a less clear separation between such genes was obtained, leaving a higher number of expression profiles as not classified. In particular, some genes, corresponding to the central low region of the plot (empty circles), showed low values of both *TNRC *and *t *statistics. This finding is not surprising, because such genes were selected on the basis of *ABCR *statistic, which may take high values even in the presence of a small difference between mean values in the two classes that cause *t *statistic to approach zero, a phenomenon that may be due to one or more outliers.

Our results confirmed the hypothesis that NPRC (identifiable by *TNRC*) may correspond to genes, whose expression profile is influenced by the presence of hidden subgroups in either class. In particular, when applied to the comparison between the semi-artificial class B, which included FL and CLL samples, and the class A, which included 14 NBC (6 heavily and 8 slightly or not stimulated samples), 13 out of 16 selected genes were able to separate almost perfectly the two hidden subgroups within either one class. In particular, the first three selected profiles in Table 2, corresponding to three clones of the same gene (*Immunoglobulin J chain*), highlighted the over-expression of all FL samples and the under-expression of all CLL samples (as indicated by the inversely-sigmoid shaped curves in Figure 3, 4 and 5), with only one exception, *i.e.*, a sample of over-expressed CLL. Interestingly, this sample was the same in the three clones (namely "CLL-52" in the original paper) [11]. J chain is a 137-amino acid protein that is synthesized in B lymphocytes and subserves 2 known functions: linking immunoglobulin monomers (IgM to pentamers, IgA to dimers) and binding polymeric immunoglobulin to secretory component [14]. Differential expression of the *Immunoglobulin J chain *gene in FL *vs *B-CLL has not been reported so far and its functional significance is unknown. The possibility that our findings reflect a statistical artifact is made unlikely by the concordant results obtained from the analysis of three different clones of the gene (Table 2). Further studies will help to better define this issue. Among the sigmoid shaped curves, which indicate the presence of two hidden sub-classes within NBC samples, the gene with the highest *TNRC *value was indicated as Gene3407X (n. 5 in Table 2) and corresponded to the *Histone deacetylase 3*. The corresponding ROC plot (Figure 7) allowed to perfectly separate Hst from Sst cells. *Histone deacetylase 3 *(HDAC3) shares functional features with HDAC1 and HDAC2. These include deacetylation of histone substrates, promoter targeted transcriptional repression and physical association with the DNA binding factor YY1 [15]. HDCA3 forms a stable complex with nuclear receptor corepressor (N-CoR) and silencing mediator of retinoic and thyroid receptors (SMRT). Beside to the direct effect on histone deacetylation, the HDAC3-N-CoR complex can exert broader functions in regulating chromatin structure. Aberrant expression and/or localization of HDCA3 have been reported in various solid tumors and myeloid leukemia [15].

*TNRC *is a supervised method of statistical analysis that, as illustrated above, may help in the identification of hidden subgroups, a task in general performed by unsupervised clustering. This latter technique has been proven to be very useful in microarray data analysis [1], because it may exploit the correlation between different gene expressions, and may identify genes that are likely to escape supervised feature selection, including *TNRC *and standard analysis based on *AUC *or *t *values. In particular, a different expression profile within a very small sub-class (*e.g.*, two or three samples) is in general hardly identifiable by supervised tests, due to their low statistical power in the presence of small sample size in either one class. Conversely, unsupervised methods tend to generate false clusters even in the presence of random values from uniform probability functions, a behavior that probably represents the major limit of such technique. Moreover, single expression profiles corresponding to NPRC may completely escape unsupervised selection method if they are weakly or not correlated to other gene expressions, but they are potentially identifiable by *TNRC*. Further studies are needed to find suitable strategies to combine unsupervised methods with supervised techniques, including our proposed approach, in microarray data analysis. Finally, the possible use of the new proposed statistics, and in particular of *ABCR*, to select genes useful for classification methods [16] should also be explored.

## Conclusion

In this paper we have illustrated a new approach for feature selection in microarray data analysis based on a combination of new and standard statistical tools exploiting the properties of ROC curves. Our method may identify both proper ROC plots, using the conventional *AUC *statistic, and NPRC, corresponding to high values of the new proposed *TNRC *parameter. *AUC *is a well known useful tool to identify over- and under-expressed genes, while *TNRC *can identify differentially expressed genes that tend to escape standard statistical analysis. We have shown that a simple visual inspection at the plot of a NPRC, selected by *TNRC*, may allow to identify hidden subclasses with potential clinical and biological insight. For these reasons, our results indicate that NPRC represent a new flexible and useful tool for the analysis of gene expression profiles from microarray experiments.

## Methods

### Data sets

We applied our new method for feature selection both to real and to simulated data sets. We selected a set of real data of gene expression by extracting 34 samples from the large data base by Alizadeh and collaborators [11], publicly available at the following web site address: . This database included 4026 gene expression profiles from a variety of 96 samples of lymphomas or non neoplastic cells. We obtained a first group (named "class A") from 14 samples of normal circulating B cells (NBC) that had been stimulated in different ways (Table 1; see also Figure 4 in the original paper). On the basis of such stimulation pattern we defined *a priori *two major subclasses within class A, *i.e.*, 6 highly stimulated and 8 slightly or not stimulated samples (Table 1). We obtained a second semi-artificial group (named "class B") of 20 heterogeneous lymphomas by pooling 9 samples of follicular lymphomas (FL) and 11 samples of chronic lymphocytic leukemia (CLL). A variable proportion of missing values for gene expression was present in each considered group. In particular, in class A the median proportion of missing values was 6.1% (range: 0.42% – 31.8%), and in class B was 4.7% (range: 0.17% – 22.5%). We estimated missing data by the method proposed by Troyanskaya and collaborators [17], using k = 12 nearest neighbor genes.

We obtained a set of artificial data bases by randomly generating normally distributed data with different means and equal variance in each class or subclass. For instance, we labeled a class of samples as "controls" and a second class of samples as "cases"; we obtained a set of not differentially expressed genes generating similar expression profiles in cases and in controls by randomly extracting simulated values from a normal standard distribution (mean = 0 and variance = 1). We obtained another set of over-expressed genes by extracting values from the same distribution, and by adopting different means for cases and controls; the mean difference (MD) between the two classes ranged from 0.5 to 3.0. Finally, we obtained a third set of differentially expressed genes, corresponding to not proper ROC curves, by splitting the cases into two subclasses (one including "under-expressed" values and one including "over-expressed" values, respectively, in comparison with the control class); in such a simulation process, we generated data from normal distributions with different means and equal variance (see as an example, Curve III in Figure 1).

Each simulated data matrix included 4000 genes. We recursively regenerated each artificial data base for 1000 times, allowing the sample size to vary between 15 to 50 in each class and the number of differentially expressed genes from 5 to 50 in each group (class or subclass, where present).

We performed all analyses by an *ad hoc *program developed in Visual Basic.net academic version (Microsoft.net framework ver. 1.1.4322), available on request. We obtained random numbers for bootstrap procedures and for data sets generation by the RAN1 algorithm [18].

### Definition of *TNRC* and *ABCR* statistics

Consider a study involving *n *subjects, classified by a binary outcome *Y *taking values in {0,1}. For example in a case-control study design, individuals with Y = 1 may be affected by a specific disease, while individuals with Y = 0 may be the unaffected controls [5]. Suppose that a variable of interest (*e.g.*, the expression level of a given gene) is measured in all the *n *subjects of the study. If *n*_0 _is the number of individuals with *Y *= 0, denote with *X*_1_, *X*_2_, ..., $X_{n_{0}}$ the values assumed by the variable of interest in this group of subjects; similarly, denote with *W*_1_, *W*_2_, ..., $W_{n_{1}}$ the values measured in the *n*_1 _individuals with *Y *= 1.

The empirical ROC curve can then be defined by considering different threshold values *c *for the variable of interest and by computing the true and the false positive fractions, denoted by TPF(*c*) and FPF(*c*), respectively, in the sample at hand [5]. It can be seen that:

$$\begin{array}{cc} \text{TPF}(c)=\frac{\sum_{j=1}^{n_{1}}I(W_{j}\geq c)}{n_{1}}, & \text{FPF}(c)=\frac{\sum_{i=1}^{n_{0}}I(X_{i}\geq c)}{n_{0}} \end{array}$$

where *I *is the indicator function providing *I*(*X*_*i *_≥ *c*) = 1 if *X*_*i *_≥ *c*, and *I*(*X*_*i *_≥ *c*) = 0 otherwise [5]. TPF corresponds to the sensitivity of a diagnostic test, while FPF corresponds to 1 – specificity. Since some of the *X*_*i *_may be equal, let {*c*_1_, ..., $c_{m_{0}}$} be the set of the *m*_0 _different values assumed by *X*_*i *_for *i *= 1, ..., *n*_0_, ordered in a decreasing way ($c_{1}>c_{2}>\cdots >c_{m_{0}-1}>c_{m_{0}}$). With these definitions, the ROC curve is given by the two dimensional graph obtained by connecting the points (FPF(*c*_*k*_), TPF(*c*_*k*_)) with straight lines, when *k *= 0, 1, ..., *m*_0_, being *c*_0 _any value greater than *X*_*i *_and *W*_*j *_for any *i *= 1, ..., *n*_0 _and any *j *= 1, ..., *n*_1_. It can be easily seen that (FPF(*c*_0_), TPF(*c*_0_)) = (0,0) whereas (FPF($c_{m_{0}}$), TPF($c_{m_{0}}$)) = (1,1).

Let *AUC*_*k *_be the partial area under an ROC curve between the consecutive abscissa points FPF(*c*_*k*-1_) and FPF(*c*_*k*_), for *k *= 1, ..., *m*_0_, computed according to the standard trapezoidal rule. The total area *AUC *under the ROC curve is then given by

$$AUC=\sum_{k=1}^{m_{0}}AUC_{k}=\sum_{k=1}^{m_{0}}\left(TPF\left(c_{k}\right)+TPF\left(c_{k-1}\right)\right)\cdot \left(FPF\left(c_{k}\right)-FPF\left(c_{k-1}\right)\right)/2$$

When TPF(*c*_*k*_) = FPF(*c*_*k*_) = *k*/*m*_0 _for *k *= 0, 1, ..., *m*_0_, every threshold *c*_*k *_is not able to provide a valid classification for the two groups of subjects, *i.e.*, the class is assigned by chance. In this case we obtain a particular ROC curve, named *chance line*, corresponding to the rising diagonal, whose partial area *AUC*_*k *_will be denoted by *A*_*k *_and is given by

$$A_{k}=\frac{2k-1}{2m_{0}^{2}}$$

It should be observed that *AUC *= 0.5 for the *chance line*.

The area *AUC *under the ROC curve gives a measure of the difference between the two distributions that generated the samples {*X*_*i*_} and {*W*_*j*_}. The greater is the value of *AUC*, the higher is the difference between the two distributions [5]. However, in some cases the ROC curve is not proper and crosses the *chance line *in one or more points. In these cases, even if the value of *AUC *is close to 0.5, the two distributions can differ significantly.

To recover these situations, the *TNRC *statistic (*TNRC *= Test for Not-proper ROC Curves) is introduced, by employing the following definition:

(1) $$TNRC=\sum_{k=1}^{m_{0}}\left|AUC_{k}-A_{k}\right|-\left|AUC-0.5\right|$$

Since in a proper ROC curve we have *AUC*_*k *_≥ *A*_*k *_for every *k *= 0, 1, ..., *m*_0_, equation (1) gives *TNRC *= 0. As a special case, this holds also for the *chance line*.

In addition, it can be easily seen that the value of *TNRC *is always non negative since

$$\sum_{k=1}^{m_{0}}\left|AUC_{k}-A_{k}\right|\geq \left|\sum_{k=1}^{m_{0}}\left(AUC_{k}-A_{k}\right)\right|=\left|AUC-0.5\right|$$

The first part of the *TNRC *statistic corresponds to the area between the ROC curve and the rising diagonal (*ABCR *= Area Between the Curve and the Rising diagonal):

(2) $$ABCR=\sum_{k=1}^{m_{0}}\left|AUC_{k}-A_{k}\right|$$

*ABCR *is a measure of the distance between the two cumulative distributions *P*_0_(*x*) and *P*_1_(*w*) that generated the samples {*X*_*i*_} and {*W*_*j*_}. This can be viewed by considering the theoretical expression of the ROC curve, which is given by $Q_{1}\left(Q_{0}^{-1}\left(z\right)\right)$ for 0 ≤ *z *≤ 1, being *Q*_0_(*x*) = 1 - *P*_0_(*x*) and *Q*_1_(*w*) = 1 - *P*_1_(*w*) [5]. In this case, *ABCR *is given by:

(3) $$\int_{0}^{1}\left|Q_{1}\left(Q_{0}^{-1}\left(z\right)\right)-z\right|dz=-\int_{+\infty }^{-\infty }\left|1-P_{1}\left(x\right)-1+P_{0}\left(x\right)\right|p_{0}\left(x\right)dx=\int_{-\infty }^{+\infty }\left|P_{1}\left(x\right)-P_{0}\left(x\right)\right|p_{0}\left(x\right)dx$$

having performed the change of variable *z *= *Q*_0_(*x*) = 1 - *P*_0_(*x*). As expected, the term at the right hand side of (3) is just the *L*_1_(*p*_0_) distance between the two distributions *P*_0_(*x*) and *P*_1_(*w*), where *p*_0 _is the probability density of the sample {*X*_*i*_}.

The distribution of *ABCR *and *TNRC *under the null hypothesis of equal gene expression in the two considered classes was estimated by 10^4 ^random permutations at different sample size, and sample mean and variance of both estimators were computed.

### Feature selection

As shown in the previous paragraph, the new described ROC parameter *ABCR *represents a measure of the distance between the distributions of gene expressions in the two considered classes. Then it may be useful to identify differentially expressed genes that may correspond both to proper and to not proper ROC curves. We performed a first step of feature selection by ranking all genes on their values of *ABCR*. The first k genes corresponding to an estimated False Discovery Rate (FDR) of 15% were retained; the analyses were also repeated using two different FDR thresholds (*i.e.*, 10% and 20%). FDR represents the proportion of gene expression profiles wrongly selected among the k top ones [12,19,20]; we obtained a conservative FDR estimation by 200 random permutations of the labels identifying either one class. Briefly, for each iteration, we computed the number ν of values higher than the *ABCR *value corresponding to the k^th ^top selected gene. The mean of ν from all permutations divided by k provided an estimate of FDR [12,20]. Finally, we estimated the probability for each gene to be included among the k ones with the highest *ABCR *statistic by the method proposed by Pepe and collaborators [8], originally used to account for multiplicity in a similar feature selection task based on another ROC parameter (*i.e.*, *pAUC*). Briefly, the probability *P*_*g*_*(k) *of each gene g to be included in such group is [8]:

*P*_*g*_(*k*) = *P *[*rank *(*g*) ≤ *k*]

We estimated *P*_*g*_*(k) *by the *bootstrap *based on 200 bootstrapped samples, which has the property to acknowledge the complex correlation between gene expression values [8]. We "jittered" each bootstrapped sample adding a randomly generated small number to each gene expression value, to avoid ties that might bias statistical estimates [8]. For this task, random values were extracted from the uniform probability function setting the range of generated values in order to preserve the original rank of not tied values.

The second step of feature selection was based on a standard ROC analysis approach: each gene selected in the previous step was classified as "under-expressed" or "over-expressed" in class B compared with class A, on the basis of the corresponding *AUC *value (values close to 0 indicating under-expression and values close to 1 indicating over-expression). Moreover, genes were also classified as corresponding to either proper or not proper ROC curves on the basis of the corresponding *TNRC *value. For both classifications an arbitrary threshold corresponding to the conventional 0.05 unadjusted p value was applied. For the first classification, the threshold value identification was based on the asymptotic normality of *AUC *and on its relation with the Mann-Whitney U statistic [5,21]. The corresponding critical value for *TNRC *was obtained by extensive permutations. For comparison purposes, the same analysis was also repeated replacing *AUC *with the Student's *t *statistic, which represents another standard tool in supervised analysis of microarrays [3,4]. Due to the non normal distribution of most gene expression profiles, which prevents the application of the Student's *t *distribution tables, statistical significance of *t *test was assessed by 5000 random permutations.

Finally, in the analysis of simulated data, for each simulation we computed the proportion of proper or uninformative curves included in the first n plots (with n = the number of genes in each simulation, corresponding to the highest *TNRC *value). Median and interquartile range (IQR) of such proportion, obtained from 1000 simulations, provided a robust estimate of FDR and of its variability, respectively. Finally, by using the same approach, the proportion of any kind of differentially expressed genes and the proportion of genes with different mean value between two classes were used to estimate FDR for *ABCR *and *AUC*, respectively.

### Interpretating the shape of a not proper ROC curve

We separated the ROC plots identified by *TNRC *into three categories, on the basis of their shape: a) sigmoid-shaped curves (*e.g.*, Curve III in Figure 1A) that may indicate the presence of a unimodal distribution of expression values in class B and a bimodal distribution in class A; b) inverse sigmoid-shaped curves (*e.g.*, Curve IV in Figure 1A) that may correspond to a bimodal distribution in class B and a unimodal distribution in class A; c) other differently shaped curves. Furthermore, we arbitrarily split each ROC curve into two parts by a vertical line crossing the centre of the plot (*i.e*, corresponding to the cut-off with a specificity value = 0.5). In sigmoid-shaped curves, such a cut-off allowed to roughly separate two alleged sub-classes of NBC, *i.e.*, samples with a higher or a lower gene expression in comparison with the median expression value of samples in class B. We evaluated the association between such sub-classes and the stimulation pattern, dichotomized into Hst and Sst, by the Fisher's exact test. Conversely, in inversely sigmoid-shaped curves, such a cut-off allowed to separate two alleged sub-classes of samples among class B, with either over- or under-expression values in comparison with NBC. We also assessed the concordance between such classification and the origin of each sample (FL or CLL) by the Fisher's exact test. The conventional unadjusted p level of 0.05 was used in this analysis.

## Authors' contributions

 SP conceived the study, performed all statistical analyses and drafted the manuscript; VP provided biological interpretation of the selected gene expression profiles; MM participated in the design and coordination of the study, and supervised statistical analyses.
